# Comparison of Consumer Rankings With Centers for Medicare & Medicaid Services Five-Star Rankings of Nursing Homes

**DOI:** 10.1001/jamanetworkopen.2020.4798

**Published:** 2020-05-14

**Authors:** Dana B. Mukamel, David L. Weimer, Yuxi Shi, Heather Ladd, Debra Saliba

**Affiliations:** 1 Division of General Internal Medicine, Department of Medicine, University of California, Irvine; 2 iTEQC Research Program, University of California, Irvine; 3 LaFollette School of Public Affairs, University of Wisconsin-Madison, Madison; 4 The Anna and Harry Borun Center for Gerontological Research, David Geffen School of Medicine, University of California, Los Angeles; 5 Veterans Administration GRECC, Los Angeles, California; 6 RAND Health, Santa Monica, California

## Abstract

**Importance:**

The Centers for Medicare & Medicaid Services (CMS) Five-Star measure for nursing homes is designed with input from expert panels about the importance of multiple quality indicators. Consumers may assign different values to these indicators, creating different 5-star ratings.

**Objective:**

To compare nursing homes’ rankings based on the CMS Five-Star measure with rankings based on consumers’ judgment about the importance of the same quality indicators.

**Design, Setting, and Participants:**

In this quality improvement study, CMS Five-Star data were linked with a measure calculated from CMS quality indicators and consumer values obtained from a national survey. Data covered the last quarter of 2016 and the first three quarters of 2017. The study included 10 676 nursing homes, comprising 69.8% of those with reported Five-Star measures. The national survey included adults, either nursing home residents or their family members who reported being familiar with the quality of care their relative received. Data analysis was performed from January 2019 to February 2020.

**Main Outcomes and Measures:**

The contingent valuation method was administered via the survey to obtain consumers’ relative values of the quality indicators, and statistical analyses were used to create the contingent valuation measure. Agreement in nursing home rankings was assessed using the Five-Star measure, which is based on weights developed by expert panels, compared with rankings based on the contingent valuation measure.

**Results:**

Among 10 676 study nursing homes with a mean (SD) of 119.4 (59.4) beds, 7845 (73.5%) were for profit, 6424 (61.8%) were part of a chain, and 8009 (75.0%) were urban. The 4310 survey respondents (mean [SD] age, 39.9 [15.6] years; 1143 [26.5%] men; 3448 [80%] white) included mostly family members (3879 participants [90.0%]). The Pearson correlation coefficient (0.65) and weighted κ statistics (0.48) indicated only moderate agreement between ranking of nursing homes’ performance by the 2 measures and disagreement on ranking for approximately one-half of the nursing homes.

**Conclusions and Relevance:**

Current nursing home report cards might not reflect consumers’ values and the relative importance consumers place on each of the quality indicators that compose the overall Five-Star measure. Quality report cards might be more relevant to consumers by augmenting the Five-Star measure with a measure reflecting consumers’ preferences. It is unknown whether these conclusions are generalizable to other report cards, such as Hospital and Home Health Compare, without conducting similar studies for these report cards.

## Key Points

**Question:**

Are nursing homes’ performance rankings the same when measured by the CMS Five-Star, a measure reflecting experts’ opinion, and when measured according to consumers’ values?

**Findings:**

In this national quality improvement study of 10 676 nursing homes, only approximately one-half of nursing homes had similar rankings with both measures.

**Meaning:**

These findings suggest that complementing the CMS Five-Star performance measure with a measure based on consumers’ values offers consumers a more complete assessment of the quality of nursing home care.

## Teaser

This quality improvement study compares nursing homes’ rankings based on the Centers for Medicare & Medicaid Services Five-Star measure with rankings based on consumers’ judgment about the importance of the same quality indicators.

## Introduction

Quality report cards that offer comparative performance rankings of health care institutions, such as hospitals, home health agencies, and nursing homes, have become ubiquitous. They aim to shift the health care system toward higher quality by influencing consumers’ choices, patient referrals, insurers’ contracting patterns, and, in response to these, institutions’ behavior.^1^ To achieve these goals, report cards have to be understood by their target audiences.^2^ Report cards that include a large number of quality indicators (QIs), which often are uncorrelated, provide mixed messages about the quality of each institution.^3,4,5,6,7^ The mixed signals tend to be confusing for consumers. In fact, decision science recognizes that less is more when it comes to making choices.^8^ To help consumers, the Centers for Medicare & Medicaid Services (CMS) added the Five-Star measure to many of its report cards. This composite measure summarizes the information included in individual QIs into 1 measure, ranging from 1 to 5 stars, with 1 indicating worst quality and 5 indicating best quality. Nursing Home Compare (NHC) introduced this measure in 2008, and it now is included in most other report cards published by CMS. An evaluation of the introduction of the NHC Five-Star measure found that it was associated with decreased demand for the lowest ranked facilities and increased demand for the highest ranked facilities.^9^ These shifts suggest that such composite measures can be effective.

Because the underlying QIs typically are not highly correlated, the Five-Star measure developed by CMS cannot be completely data driven. Instead, CMS relies on other considerations, including the advice of expert panels composed of clinicians, economists, epidemiologists, and others. The design of the Five-Star measure reflects the expertise and judgments of these panels about the relative importance of each component QI.^10^

Designing the Five-Star measure on the basis of expert judgment introduces the possibility of alternative designs that are based on different judgments of the relative importance of the QIs. Such designs may lead to different rankings of institutions than the Five-Star measure. In particular, we are interested in designs that consider the values and preferences of nursing home residents and their family members. Residents’ views about the attributes of quality may differ from the views of experts; therefore, a composite measure that reflects the relative importance residents assign to each QI will likely differ from a composite measure reflecting the relative importance that experts assign to the same QIs. Mukamel et al^11^ have shown that individuals, when given the opportunity to construct their own, personal, individual composites, choose the same nursing home as they would on the basis of the Five-Star Nursing Home Compare measure only one-third of the time.

We present an analysis that compares the Five-Star NHC measure to an alternative, consumer-based measure developed by Weimer et al.^12^ The Five-Star NHC measure uses CMS weights, based on technical experts panels’ advice, to combine QIs for staffing, facility citations from annual nursing home regulatory surveys, and clinical quality measures. The consumer measure combines the QIs using weights derived from a survey of a large, national sample of residents of nursing homes and family members of nursing home residents who are familiar with residents’ care and facility quality. Development of the alternative consumer measure used the contingent valuation method (CVM) to determine the relative value (weights) individual residents and families assign to each QI.

## Methods

This study was approved by the University of California, Irvine, institutional review board. An information sheet was provided to respondents before administering the survey, and oral informed consent was obtained.

### Methodological Issues in Comparing the Five-Star and CVM Measures

Two issues require consideration. First, when the CVM measure was developed, it included only 7 of the 16 QIs included in the Five-Star measure at the time. Therefore, when comparing the measures, one needs to separate the difference between the measures resulting from differing QIs and differing weights used to create the composite measures. It is the latter that we are interested in identifying. We accomplished this by recalculating a revised Five-Star measure based on the same QIs used in the CVM measure but using the CMS Five-Star method.^10^ This approach keeps the QIs constant, while changing the weights; therefore, any differences between the revised Five-Star and the CVM measures can be attributed to the weights, or preferences, of the experts vs consumers.

The second issue is related to the timing of the data. The CVM weights were obtained in a national survey administered during the third quarter of 2017.^12^ Therefore, to align these weights, which reflect survey respondents’ contemporaneous experience with care in the nursing home with the measured CMS QIs and Five-Star, all data in this analysis were obtained for the same period.

### Data and Sample

The initial data set included all 15 288 Medicare- and Medicaid-certified nursing homes in the US in the third quarter of 2017 with nonmissing Five-Star measures. We excluded 4082 nursing homes (26.7%) for missing the minimum data set (MDS) and claims-based quality data and 530 nursing homes (3.5%) for missing health inspections and staffing indicators. The final sample included 10 676 (69.8%) facilities.

All data were downloaded from the CMS NHC archived data website.^13^ For small facilities, for which CMS does not publicly report MDS-based quality measures, we obtained the quality measures directly from CMS.

### Variables

#### CMS Five-Star Measures

Nursing home characteristics were obtained from the CMS NHC website^13^ and from Long-Term Care Focus.^14^ As discussed already, we calculated a revised Five-Star based on published QIs from the third quarter of 2017 using the CMS method and obtained the CMS 5-Star measure published in January 2018, which was also based on data for the third quarter of 2017.

#### CVM Measures

To develop the CVM measures in a manner analogous to the CMS Five-Star measure, we first created the individual QIs using the CMS data and method for estimating each QI. We then used the weights developed from the CVM consumers’ survey to create the composite measure that reflected consumers’ values. Our description of the 2 steps follows.

First, we calculated individual QIs. The CVM includes measures based on information from the MDS, Medicare claims data, and the annual regulatory survey of nursing homes. The MDS-based QIs (the clinical quality measures) included percentage of long-stay residents with pressure sores, percentage of long-stay residents whose need for assistance with activities of daily living increased, percentage of long-stay residents who report moderate-to-severe pain, and percentage of short-stay residents who report moderate-to-severe pain. In accordance with the CMS, we calculated the mean for each QI over the last 4 quarters ending with the third quarter of 2017. If the denominator was less than 20, then the QI was set to missing.

The claims-based QIs included rehospitalization and successful discharge to the community. These were obtained directly from the most recent data published by CMS: calendar year 2017 data for rehospitalization and the year ending in October 2016^10^ for successful discharge to the community.

Two survey-based QIs included health inspections and total nurse staffing hours per resident per day. The CMS defines health inspections as the total number of deficiencies, measured as a moving weighted mean over the preceding 3 years, with the highest weight given to the most recent year. Deficiencies are also weighted by their scope and severity.^10^ Total nurse staffing hours per resident per day are adjusted for resident case mix and include registered nurses, licensed practical nurses, and certified nurse assistants.^10^ The Five-Star bases these QIs on the annual state regulatory survey data. We obtained data for the period ending in the third quarter of 2017.

Next, we calculated a continuous CVM measure. The CVM is described in detail in Weimer et al.^12^ Here we provide a brief summary. We surveyed either nursing home residents or their family members who reported having knowledge of the quality of care their relative received. We presented survey respondents with scenarios asking them to trade off specific improvements in quality attributes for longer travel time to visit their loved one. For example, a typical question posed was “Would you be willing to travel an additional 30 minutes to visit your relative if the nursing home provided better care, such that 10% fewer residents had pressure ulcers?” (for scenario details, see Weimer et al^12^). We randomly assigned selected travel distances and levels of quality improvement defining the tradeoff that the respondent faced. Each individual responded to a different scenario, resulting in large samples for each improvement type (eg, lower rates of pressure sores). These were analyzed statistically to provide the weight for each QI. These weights were then used to create a continuous CVM measure.

We calculated 2 CVM measures, 1 based on the quality measures (MDS and claims QIs) only, which we compared in further analyses to the revised Five-Star Quality Measures component, and 1 based on all QIs, which we compared with the overall revised Five-Star measure. In sensitivity analyses, we compared the CVM with the CMS Five-Star published measures that included all 16 QIs.

Finally, we calculated a 5-step CVM measure. To compare the CVM measure with the CMS Five-Star measure, we converted the continuous CVM measures to a 5-step scale, using the same empirical distribution as exhibited by the revised Five-Star measures. For example, 28% of nursing homes in our sample had 5 stars according to the overall revised CMS Five-Star measure. We therefore assigned the top 28% nursing homes in the continuous CVM distribution to the top of the 5-step scale CVM measure.

### Statistical Analysis

We calculated descriptive statistics for the nursing homes included in our study sample and used χ^2^ and 2-sided *t* tests to compare these nursing homes with those excluded from the analyses due to missing data. We then compared the measures in 2 ways. Statistical significance was set at *P* < .01.

First, we calculated the Pearson correlation coefficient between the CVM and the revised Five-Star measures. Second, we compared the agreement between the revised Five-Star and the CVM measures on the rank ordering of nursing homes into 5 categories from worst (1) to best (5). We sorted all nursing homes by the revised Five-Star measure and independently by the CVM 5-step scale and cross-tabulated them. We calculated a linear weighted κ value, which measures the overall agreement between the 2 measures. The weights for each cell are a function of the distance between the CVM 5-step and the CMS revised Five-Star cells of same rank. If both methods assign a nursing home to the same cell (eg, 5/5, 4/4) the κ weight is the highest. The weight declines as the distance between the cells increases and is least for a 5/1 or 1/5 assignment.^15^ In addition to the weighted κ, we also present and discuss the full cross-tabulation showing the correspondence between the revised Five-Star and the CVM 5-step scale measures. All calculations were done with Stata statistical software version 14.2 (StataCorp). Data analysis was performed from January 2019 to February 2020.

## Results

The study included 10 676 nursing homes, comprising 69.8% of those with reported Five-Star measures. Table 1 presents descriptive statistics. Of the study nursing homes, 7845 nursing homes (73.5%) are for profit, 6424 nursing homes (61.8%) are part of a chain, and 8009 nursing homes (75.0%) are urban. They are substantially different from the 4612 facilities (30.2%) excluded from the analyses for all but percentage of long-stay residents with pressure sores (mean [SD], 5.6% [3.4%] vs 5.5% [4.5%]) and percentage of short-stay residents who were rehospitalized (mean [SD], 22.0% [5.5%] vs 21.9% [7.8%]). In general, they tend to be larger (as expected according to the CMS method inclusion criteria), with a mean (SD) of 119.4 (59.4) vs 77.1 (53.1) beds, and have better QI scores than the excluded facilities (nursing homes with a rating of 5, 5489 [51.4%] vs 1931 [42.4%]).

**Table 1.** Comparison of Study Nursing Homes With Nonstudy Nursing Homes

Characteristic	Nursing homes, No. (%)		*P* value^c^
	Study (n = 10 676)^a^	Nonstudy (n = 4612)^b^	
CMS Five-Star quality ratings			
1	374 (3.5)	386 (8.4)	<.001
2	986 (9.2)	628 (13.6)	
3	1682 (15.8)	741 (16.1)	
4	2348 (22.0)	865 (18.8)	
5	5286 (49.5)	1992 (43.2)	
CMS Five-Star quality ratings based on 7 quality indicators			
1	271 (2.5)	322 (7.1)	<.001
2	1286 (12.1)	899 (19.8)	
3	1615 (15.1)	684 (15.0)	
4	2015 (18.9)	714 (15.7)	
5	5489 (51.4)	1931 (42.4)	
CMS Five-Star overall ratings			
1	1230 (11.5)	688 (14.9)	<.001
2	2158 (20.2)	895 (19.4)	
3	1811 (17.0)	757 (16.4)	
4	2487 (23.3)	986 (21.4)	
5	2990 (28.0)	1286 (27.9)	
CMS Five-Star overall ratings based on 7 quality indicators			
1	1092 (10.2)	501 (11.1)	<.001
2	2237 (21.0)	878 (19.5)	
3	1886 (17.7)	747 (16.6)	
4	2531 (23.7)	992 (22.0)	
5	2930 (27.4)	1390 (30.8)	
Long-stay residents, mean (SD), %			
With pressure sore	5.6 (3.4)	5.5 (4.5)	.14
Whose need for aid with activities of daily living limitations has increased	14.7 (6.0)	15.5 (7.1)	<.001
With pain	5.0 (4.9)	6.9 (6.6)	<.001
Short-stay residents, mean (SD), %			
With pain	12.7 (9.3)	16.1 (12.0)	<.001
Who were rehospitalized	22.0 (5.5)	21.9 (7.8)	.60
Who were successfully discharged to the community	53.7 (10.8)	57.4 (11.2)	<.001
Case mix–adjusted total nursing per resident day, mean (SD), No.	3.8 (0.7)	4.2 (1.2)	<.001
Health deficiencies, mean (SD), No.	7.7 (6.8)	7.1 (6.9)	<.001
Certified beds, mean (SD), No.	119.4 (59.4)	77.1 (53.1)	<.001
Type of facility			
Hospital based	198 (1.9)	487 (10.6)	<.001
For profit	7845 (73.5)	2850 (61.8)	
Part of a chain	6424 (61.8)	2181 (49.6)	
Changed ownership during the last 12 mo	263 (2.5)	140 (3.0)	.04
Residents, mean (SD), %			
Medicare recipients	15.3 (10.6)	11.7 (19.2)	<.001
Medicaid recipients	58.7 (20.5)	61.6 (28.1)	<.001
Nursing home is in an urban area	8009 (75.0)	2723 (59.0)	<.001
Nursing home location			
Northeast	2078 (19.5)	493 (10.7)	<.001
Midwest	3077 (28.8)	1960 (42.5)	
South	3901 (36.5)	1421 (30.8)	
West	1620 (15.2)	733 (15.9)	

Abbreviation: CMS, Centers for Medicare & Medicaid Services.

^a^ Chain and payer data were available for only 10 392 nursing homes.

^b^ Most data loss was due to missing quality measures data from small nursing homes.

^c^ *P* values are for the hypothesis that characteristics of the study nursing home and the nonstudy nursing homes are the same.

The Pearson correlation coefficient between the MDS and claims components of the CVM and the CMS revised Quality Measure Five-Star was 0.61, and the correlation coefficient between the full CVM, including all QIs and the CMS revised overall Five-Star measures, was 0.65. The correlation coefficient can assume values from 0, indicating no agreement at all (random assignment), to 1, indicating perfect agreement. Our findings indicate a moderate correlation.^16^

In total, we surveyed 4310 adults (mean [SD] age, 39.9 [15.6] years; 1143 [26.5%] men; 3879 family members [90.0%]; 3448 white [80.0%]). Table 2 and Table 3 show the assignment to the 5 levels and agreement between the revised Five-Star and the CVM measures. Table 2 compares the MDS and claims components of the 2 measures in terms of percentage of nursing homes in each category. On the diagonal, the numbers present the percentage of nursing homes for which both measures agree. The most agreement is on the highest quality assignment (the 5/5 cell, with 37.5% of facilities). As we move off the diagonal, the percentage of facilities decreases, indicating less agreement on assignment to any off-diagonal cell, although the sum of the percentage of facilities in the off-diagonal often exceeds the percentage of nursing homes on the diagonal. Furthermore, as we move toward lower quality (the 1/1 cell) the percentage of facilities declines steeply to 0.7%.

**Table 2.** Comparison of the Revised CMS Five-Star and CVM 5-Step Scale Composite Quality Ratings: Quality Component Only^a^

CVM 5-step rating	Revised CMS Five-Star rating, % of nursing homes in each category					
	1 (Lowest quality)	2	3	4	5 (Best quality)	Total^b^
1 (Lowest quality)	0.7^c^	1.6	0.7	0.4	0.2	3.5
2	0.9	3.0^c^	2.6	1.6	1.1	9.2
3	0.7	3.9	3.7^c^	3.7	3.8	15.8
4	0.1	2.9	4.6	5.5^c^	8.9	22.0
5 (Best quality)	0.0	0.8	3.5	7.7	37.5^c^	49.5
Total	2.5	12.0	15.1	18.9	51.4	100.0

Abbreviations: CMS, the Centers for Medicare & Medicaid Services; CVM, contingent valuation method.

^a^ The revised Five-Star measures include the same quality indicators as the CVM measures.

^b^ Numbers may not total 100 because of rounding.

^c^ Percentage of nursing homes for which both measures agree.

**Table 3.** Comparison of the Revised CMS Five-Star and CVM 5-Step Scale Composite Overall Quality Ratings^a^

CVM 5-step rating	Revised CMS Five-Star rating, % of nursing homes in each category					
	1 (Lowest quality)	2	3	4	5 (Best quality)	Total^b^
1 (Lowest quality)	4.9^c^	4.6	1.3	0.7	0.1	11.6
2	3.5	7.4^c^	5.1	3.3	0.9	20.2
3	1.2	4.0	4.3^c^	5.1	2.4	17.0
4	0.5	3.3	4.8	7.9^c^	6.9	23.4
5 (Best quality)	0.1	1.6	2.2	6.8	17.3^c^	28.0
Total^b^	10.2	20.9	17.7	23.8	27.6	100.0

Abbreviations: CMS, the Centers for Medicare & Medicaid Services; CVM, contingent valuation method.

^a^ The revised Five-Star measures include the same quality measures as the CVM measures. Note that the composite measures include all quality indicators (the quality measures in Table 2, and the staffing and health deficiencies). The 5-Star composite weighs them using the CMS method. The CVM 5-Step measure weighs them using the CVM weights.

^b^ Numbers may not total 100 because of rounding.

^c^ Percentage of nursing homes for which both measures agree.

Table 3 presents the same information for the revised overall CMS Five-Star and CVM 5-Step measures. The findings are generally similar with 2 noteworthy exceptions. The highest percentage of facilities in the 5/5 cell is only one-half as large, at 17.3% of all nursing homes, and there is more agreement between the CVM and the revised CMS Five-Star measures in the lower quality levels, particularly for the 1/1 cell at almost 5% compared with less than 1% for the quality measures only measures, shown for the quality measures comparison.

The weighted κ value calculated for the quality measures comparison of Table 2 and Table 3 was 0.44, and that for the revised overall measure was 0.48. The κ value can range from −1 to 1, with 0 indicating random assignment and 1 indicating perfect agreement. Our findings indicate only moderate agreement between the revised CMS Five-Star and the CVM 5-Step measure.^15^

Findings of the sensitivity analysis comparing the CVM with the published Five-Star measures were very similar and, therefore, are not presented or discussed here. They are available as the eTable in the Supplement.

## Discussion

This study found that the rankings obtained from a consumer-driven approach had only moderate agreement with rankings obtained from the traditional, technical-expert informed approach currently used in NHC. This consumer-driven approach uses a composite measure that has been demonstrated to be a feasible alternative to the CMS Five-Star.^12^

To assign these ratings, we used the same sample of nursing homes and the same underlying data about quality measures where possible. We compared a revised Five-Star (which keeps the number of QIs equal to those included in our CVM measure) and the CVM measure in 2 ways. We found that the Pearson correlation coefficient between the 2 measures ranged from 0.61 for the component based on the quality measures (MDS and claims) only to 0.65 for the overall measures. These values are typically viewed as moderate agreement.^16^ We also found weighted κ values ranging from 0.44 to 0.48. These values also indicate only moderate agreement.

What are the policy implications of these findings? The literature^3,4,5,6,7^ has demonstrated that quality measures tend to be uncorrelated, and, hence, composite measures have to be constructed on the basis of opinions or preference weights. Different stakeholders have different views on whose preferences should determine the weights in constructing such composite measures. The practice to date, spearheaded by CMS, is to use experts’ opinion to form such weights. We were motivated to develop this alternative by the notion that consumer preferences deserve consideration as well. The comparison data we present suggest that ranking of nursing homes based on expert informed weights coincides with ranking based on consumer informed weights only about one-half of the time. This suggests that a report card should include both types of summary measures, the one based on experts’ opinion and that based on experienced consumers’ preferences. Providing both would allow patients to compare them and, to the degree that they differ, decide whether they are more comfortable with the advice of an expert or another consumer. One could imagine that, given the choice, consumers and purchasers would prefer to select nursing homes where both the expert-weighted and the consumer-weighted approach assign the highest rating.

### Limitations

We should note several limitations of this study. Because of the way the CVM measure is constructed,^12^ the QIs do not replicate completely those included in the CMS Five-Star composite. We accounted for this by comparing the CVM measure with a revised Five-Star measure that we constructed to be comparable in terms of the QIs, thus allowing the differences to be attributed to differences in opinions between the experts and consumers. The eTable in the Supplement, which shows the results of the comparison with the full Five-Star, shows very similar findings. Thus, despite this limitation, our findings seem robust. However, before CVM measures can be offered in report cards, preference elicitation exercises for additional QIs need to be conducted if the CVM measures are to be as comprehensive as the Five-Star.

Our study excluded 30.2% of nursing homes, mostly because of missing QI data. Because these data are more likely to be missing for smaller nursing homes, our results do not generalize to this group of nursing homes.

Finally, one should consider the question of whether report cards for other institutions, such as Hospital Compare and Home Health Compare, might similarly benefit from adding the consumer perspective to their Five-Star measures. Our findings do not generalize to these report cards. One would require studies similar to that by Weimer et al^12^ and the analysis we present here to answer this question. It should be noted, however, that the inclusion of the Consumer Assessment of Healthcare Providers and Systems (CAHPS) survey measures in both the hospital and the home health report cards does not meet the need to offer a composite measure that is based on consumer values. The Consumer Assessment of Healthcare Providers and Systems surveys provide quality measures reflecting quality domains that consumers can easily evaluate and appreciate. However, they do not address the question that the CVM does, which is the question of the relative importance and value that consumers place on these and other quality measures when they are combined into a composite measure. Therefore, the Consumer Assessment of Healthcare Providers and Systems and the CVM each plays a different, albeit important, role and offers a different contribution to the report card.

## Conclusions

The analysis we present here provides both good and bad news. It validates the CMS Five-Star measure from a consumer perspective in approximately one-half of the cases, but also suggests that much useful information would result from including consumers’ perspectives.

## Notes

Supplement. **eTable.** Comparison of CMS Five-Star and CVM 5-Step Scale Composite Quality Rating
