# Supplementary material for: Comparison of Consumer Rankings With Centers for Medicare & Medicaid Services Five-Star Rankings of Nursing Homes
Source: JAMA Netw Open. 2020 May 14;3(5):e204798. doi: 10.1001/jamanetworkopen.2020.4798 (PMC7225897; doi:10.1001/jamanetworkopen.2020.4798)
Supplement: Supplement. — eTable. Comparison of CMS Five-Star and CVM 5-Step Scale Composite Quality Rating [file jamanetwopen-3-e204798-s001.pdf]

Supplementary Online Content

Mukamel DB, Weimer DL, Shi Y, Ladd H, Saliba D. Comparison of consumer rankings with Centers for Medicare & Medicaid Services Five-Star rankings of nursing homes. *JAMA Netw Open*. 2020;3(5):e204798. doi:10.1001/jamanetworkopen.2020.4798

eTable. Comparison of CMS Five-Star and CVM 5-Step Scale Composite Quality Rating

This supplementary material has been provided by the authors to give readers additional information about their work.

© 2020 Mukamel DB et al. *JAMA Network Open.*

eTable: Comparison of CMS Five-Star and CVM 5-Step Scale Composite Quality Rating*

Quality component of the CMS Five-Star and CVM 5-Step Scale Quality Rating						
CVM 5-Step Quantiles	CMS Five-Star Quantiles, Percent of nursing homes in each category					**Total**
	1=lowest quality	2	3	4	5=best quality	
1=lowest quality	**1.1**	1.1	0.8	0.3	0.1	**3.5**
2	1.3	**2.6**	2.5	1.8	1.0	**9.2**
3	0.7	2.8	**4.7**	4.7	3.0	**15.8**
4	0.3	1.9	4.4	**6.6**	8.8	**22.0**
5=best quality	0.1	0.8	3.3	8.7	**36.5**	**49.5**
**Total**	**3.5**	**9.2**	**15.8**	**22.2**	**49.5**	**100.0**
Overall CMS Five-Star and CVM 5-Step Scale Overall Rating						
CVM 5-Step Quantiles	CMS Five-Star Quantiles, Percent of nursing homes in each category					**Total**
	1=lowest quality	2	3	4	5=best quality	
1=lowest quality	**5.7**	4.1	1.5	0.4	0.1	**11.5**
2	3.8	**7.4**	5.1	3.3	0.9	**20.2**
3	1.2	4.0	**4.5**	4.9	2.3	**17.0**
4	0.7	3.3	4.2	**7.9**	7.0	**23.3**
5=best quality	0.1	1.1	1.8	6.7	**17.7**	**28.0**
**Total**	**11.5**	**20.2**	**17.0**	**23.3**	**28.0**	**100.0**

*Note that the composite measures include all quality indicators (the QMs in tables A1, and the QMs, staffing and health deficiencies in table A2). The 5-Star composite weighs them using the CMS methodology. The CVM 5-Step measure weighs them using the CVM weights.

© 2020 Mukamel DB et al. *JAMA Network Open.*
